# Exploration of the impact of the COVID-19 pandemic on the mental health of home health care workers in Japan: a multicenter cross-sectional web-based survey

**DOI:** 10.1186/s12875-022-01745-4

**Published:** 2022-05-26

**Authors:** Jun Hamano, Hirokazu Tachikawa, Sho Takahashi, Saori Ekoyama, Hiroka Nagaoka, Sachiko Ozone, Shoichi Masumoto, Takahiro Hosoi, Tetsuaki Arai

**Affiliations:** 1 Division of Clinical Medicine, Faculty of Medicine, University of Tsukuba, 1-1-1 Tennoudai, Tsukuba, Ibaraki 305-8575 Japan; 2 Department of Disaster and Community Psychiatry, Faculty of Medicine, University of Tsukuba, Tsukuba, Japan; 3 University of Tsukuba Hospital, Tsukuba, Japan; 4 Department of Family Medicine, Faculty of Medicine, General Practice and Community Health, University of Tsukuba, Tsukuba, Japan; 5 Department of General Medicine, Tsukuba Central Hospital, kamikashiwada 4-58-1, Ushiku, Ibaraki 300-1232 Japan

**Keywords:** Home health care workers, COVID-19 pandemic, Fear of COVID-19 scale, Hospital Anxiety and Depression scale, Assessment of Interprofessional Team Collaboration Scale-II

## Abstract

**Background:**

The COVID-19 pandemic has caused home health care workers (home-HCWs) to experience anxiety. The mental health of home-HCWs and related factors during the COVID-19 pandemic have not been clarified; therefore, we aimed to investigate the status and associated factors of fear of COVID-19 infection, anxiety, and depression among home-HCWs in Japan.

**Methods:**

We conducted a multicenter cross-sectional web-based anonymous survey of home-HCWs in August 2021, during the fifth wave of the pandemic in Japan. We surveyed members of facilities that provided home visit services during the COVID-19 pandemic. We measured the Japanese version of the Fear of COVID-19 scale (FCV-19S-J) and the Hospital Anxiety and Depression scale (HADS) as objective variables, and the Japanese version of the Assessment of Interprofessional Team Collaboration Scale-II (J-AITCS-II) as an explanatory variable.

**Results:**

A total of 328 members of 37 facilities responded to the survey, and we ultimately analyzed 311 participants. The most frequent occupation was nurse (32.8%), followed by doctor (24.8%) and medical office staff (18.0%). The mean score of the FCV-19S-J was 16.5 ± 5.0 (7.0 – 31.0), and the prevalences of definitive anxiety and depression were 7.4% and 15.7%, respectively. Multivariate regression analysis revealed that the J-AITCS-II teamwork subscale was significantly negatively associated with FCV-19S-J, HADS-anxiety, and HADS-depression (β = -0.171, *p* = 0.004; β = -0.151, *p* = 0.012; β = -0.225, *p* < 0.001, respectively). Medical office staff showed significant positive associations with FCV-19S-J and HADS-depression (β = 0.219, *p* = 0.005; β = 0.201, *p* = 0.009, respectively), and medical social workers with HADS-anxiety and HADS-depression (β = -0.166, *p* = 0.011; β = -0.214, *p* < 0.001, respectively) compared with doctors. The unmet support need for expert lectures on COVID-19 was significantly positively associated with FCV-19S-J (β = 0.131, *p* = 0.048), and the unmet support need for support systems for psychological stress and emotional exhaustion was significantly positively associated with HADS-anxiety (β = 0.141, *p* = 0.022).

**Conclusions:**

Fear of COVID-19 infection and depression of nurses, medical office staff, and other occupations was significantly higher than those of doctors. These findings suggest that non-physicians were more likely to be fearful and depressed during the COVID-19 pandemic; thus, it is necessary to tailor mental health support based on occupation in the home care setting.

## Background

Home health care workers (home-HCWs) for community-dwelling patients play an indispensable role in supporting patients and their family members during the COVID-19 pandemic. Home-HCWs, including home visiting doctors, nurses, medical social workers, care workers, and medical office staff, spend more time in contact with patients and their families and are at potential risk of COVID-19 infection because appropriate triage and zoning are more difficult at home than in the hospital setting.

A recent study reported home-HCWs received inadequate support during the pandemic and that this was not recognized [1]. In addition, home-HCWs were exposed to high stress even before the COVID-19 pandemic, and the COVID-19 pandemic has caused them to feel anxiety. Strengthening of the support system for home-HCWs has therefore been proposed [1–3]. A recent systematic review and cross-sectional studies reported the impact of the COVID-19 pandemic on the mental health of healthcare workers in acute care hospitals, related factors, and the support they needed [4–6]. Quadros et al. reported the prevalence of fear of COVID-19 infection to be 18.1–45.2% in the general population, and that working in healthcare was a risk factor [7]. Moreover, a nationwide study in Poland reported that frontline medical workers and medical professionals who were made to take a secondment to fight the COVID-19 pandemic had significantly higher prevalence of mental disorders compared to non-medical professionals [8].

Hao et al. reported the prevalences of depression and anxiety during the COVID-19 pandemic to be 24.1% and 28.6%, respectively, in acute care hospitals, with females and nurses having particularly high rates of depression [9]. In addition, Hao et al. reported that the occupational attribute of medical staff was a protective factor for mental health compared to non-medical staff [9]. However, the status of fear of COVID-19 infection and prevalences of anxiety and depression during the COVID-19 pandemic among home-HCWs and their related factors have not been clarified [10].

Clarification of the fear of COVID-19 infection, mental health, and unmet support needs of home-HCWs could improve the working environment during the COVID-19 pandemic and promote preparedness for future pandemics in the home care setting. The present study aimed to investigate the status and associated factors of fear of COVID-19 infection and the prevalences of anxiety and depression among home-HCWs in Japan. Based on several previous studies and discussion among the authors [1, 4–7, 9, 10], we proposed the following hypotheses: (1) there was a difference in fear of COVID-19 infection among the home-HCWs; (2) there was a difference in the prevalence of anxiety and depression of the home-HCWs during the COVID-19 pandemic; and (3) there were differences of unmet support needs of home-HCWs during the COVID-19 pandemic.

## Methods

### Study design and setting

A multicenter cross-sectional web-based anonymous survey of home-HCWs was conducted in August 2021, during the fifth wave of the pandemic in Japan, using the SurveyMonkey platform [11]. SurveyMonkey is an online service that facilitates sharing surveys via email, smartphone applications, and social media platforms such as Facebook and Twitter. In the fifth wave of COVID-19 in Japan, about 900,000 people were infected with COVID-19, the estimated death rate for those over 65 years old was 2.5%, and the rate of the people who had completed two doses of the vaccine was about 64% [12]. As a result, the Japanese government declared a state of emergency and urged its citizens to refrain from going out unnecessarily.

This study was conducted under the ethical standards of the Declaration of Helsinki and the Ethical Guidelines for Epidemiological Research issued by the Ministry of Health, Labour and Welfare of Japan. The Institutional Review Board of the University of Tsukuba approved this study (No. 1651).

### Participants

We recruited patients from facilities that provided home visit services during the COVID-19 pandemic based on previous studies in Japan [13–15]. We asked the director of each facility to ask their staff to respond to a web-based anonymous survey. We did not ask how many staff were candidates for this survey at each facility. We explained the purpose of this study and asked for informed consent to participate in this study using our web page. Only staff who agreed to participate in the study were included. Exclusion criteria for participants were: duplicate responses from the same IP address, and no response.

### Participant characteristics

We asked each participant about background characteristics: sex, age, type of occupation, years of experience in the occupation, coronavirus vaccination status, experience of training in wearing personal protective equipment (PPE), living with family or not, and risk of coronavirus infection. We also asked about the availability of information and support, and the impact and difficulties of the COVID-19 pandemic on home care service such as increase in workload and difficulty of communication with colleagues.

A recent systematic review reported that interprofessional collaboration is related to job stress of health care workers [16]; therefore, we evaluated the perception of interprofessional collaboration of each participant using the subscale of the Japanese version of Assessment of Interprofessional Team Collaboration Scale-II (J-AITCS-II) [17]. AITCS-II was developed as a shortened version of the Assessment of Interprofessional Team Collaboration Scale, a measurement tool developed for evaluating collaboration within teams across various practice settings and the integration of patient involvement as part of team practice [18]. Yamamoto and Haruta confirmed the validity and reliability of the two subscales (patient-centered collaborative care and teamwork) of J-AITCS-II [17] in healthcare professionals. Based on previous studies and discussion among the authors, we measured the subscale of teamwork in healthcare professionals as a participant characteristic [5, 19, 20].

### Questionnaire

In the absence of specific and validated instruments for evaluating the support currently received and the support needs of home-HCWs during the COVID-19 pandemic, we developed an original questionnaire based on data from previous studies and discussion among the authors of this study [5, 19, 20]. An example of a question and answer about support currently received is: “I have been provided with expert on-site infection control guidance: yes/no”, and an example of a question and answer about support needs is “The need for experts to provide on-site infection control guidance”, which was answered using a 6-point Likert scale answer (“not needed at all” “not needed” “little need” “some need” “needed”, and “very much needed”).

### Measurements

#### Japanese version of the fear of COVID-19 scale

The Fear of COVID-19 Scale (FCV-19S) was developed by Ahorsu et al. to measure anxiety and fear of COVID-19 using a seven-item self-administered scale [21]. The FCV-19S-J was developed and confirmed for reliability and validity by Midorikawa et al. [22]. The response options were “strongly disagree,” “disagree,” “neither agree nor disagree,” “agree,” and “strongly agree”. The minimum score possible for each question is 1 (strongly disagree) and the maximum is 5 (strongly agree). The total score is calculated by adding up each item score (ranging from 7 to 35). The higher the score, the greater the fear of COVID-19.

#### Hospital anxiety and depression scale

The Japanese version of the Hospital Anxiety and Depression Scale (HADS) was confirmed for validity and reliability [23]. The HADS comprises a total of 14 items, including seven that measure recent anxiety and another seven that measure recent depression [24], and each question is answered by four possible responses. Higher scores denote more severe anxiety or depression experienced recently.

### Statistical analysis

After calculating the sum of the seven items in FCV-19S-J, we then calculated the HADS and analyzed the distributions of other factors. We defined support as unmet when the participant answered that support was “very much needed” or “needed” but had not been received. Subsequently, we performed multivariate regression analysis of the FCV-19S-J, the score of HADS-anxiety, and HADS-depression with 10 variables: sex, age, type of occupation, score of J-AITCS-II teamwork subscale, increase in workload, unmet support needs (experts to provide on-site infection control guidance, consultation with infection control experts online or by phone, expert lectures on COVID-19, distribution system for PPE by national or local government, expert support systems for psychological stress and emotional exhaustion), based on a previous study and discussion among the authors [7, 9, 10, 20, 25–27].

Significance was *p* < 0.05 and all analyses were carried out using SPSS-J software (ver. 27.0; IBM, Tokyo, Japan).

## Results

A total of 328 participants from 37 facilities responded to the survey. We excluded 17 participants whose responses could not be registered. No participant responded more than once from the same IP address. Thus, we analyzed 311 participants. Characteristics of the participants are summarized in Table 1. The most frequent age group was 40–49 (37.6%), followed by 30–39 (27.3%), and 69.5% were women. The type of occupation was most frequently nurse (32.8%), followed by doctor (24.8%) and medical office staff (18.0%). Experience in the occupation was most frequently greater than 20 years (28.3%). Coronavirus vaccines had been received at least once by 94.5% of the participants, and 85.2% had experienced a great deal or quite a lot of stress due to COVID-19 during the previous month.

**Table 1:** Participants’ background characteristics

	*n* = 311	%
Age		
20's	21	6.8
30's	85	27.3
40's	117	37.6
50's	67	21.5
60's	17	5.5
70's	3	1.0
Sex		
male	94	30.2
female	216	69.5
Contact with patients in daily practice		
Yes	236	75.9
Type of occupation		
Doctor	77	24.8
Nurse	102	32.8
Medical social worker	21	6.8
Medical office staff	56	18.0
Other	54	17.4
Years of experience		
≤ 2 years	38	12.2
3–4 years	35	11.3
5–9 years	65	20.9
10–19 years	84	27.0
≥ 20 years	88	28.3
Living with family		
Yes	256	82.3
Living with family under 15 years old		
Yes	140	45.0
Living with family over 65 years old		
Yes	56	18.0
Have participated in training on use of personal protective equipment		
Yes, before January 2020	63	20.3
Yes, after February 2020	81	26.0
No	164	52.7
Have received coronavirus vaccine at least once		
Yes	294	94.5
Were you ever at risk of contracting COVID-19?		
Yes	176	56.6
Were your family or friends ever at the risk of contracting COVID-19?		
Yes	135	43.4
During the past month, how stressed have you been about COVID-19?		
Not at all/Not much	24	7.7
Neither	19	6.1
A great deal/Quite a bit	265	85.2
How do you feel about your own health condition compared to before the COVID-19 pandemic?		
No change	250	80.4
Better	17	5.5
Worse	41	13.2
The COVID-19 pandemic has increased my workload		
Absolutely disagree/Disagree/Somewhat disagree	56	18.0
Absolutely agree/Agree/Somewhat agree	248	79.7
The Japanese version of Assessment of Interprofessional Team Collaboration Scale-II (*n* = 293)		
mean ± standard deviation	35.4 ± 5.7	
median (min, max)	36.0 (9.0, 45.0)	

Table 2 shows the received support, support needs, and unmet support needs of home-HCWs during the COVID-19 pandemic. The most unmet support need was for experts to provide on-site infection control guidance (39.2%), followed by systems to consult with infection control experts online or by phone (38.3%), and systems to support psychological stress and emotional exhaustion (37.9%). There were significant differences in support needs regarding expert lectures on COVID-19 (*p* = 0.002) and distribution systems for PPE by national or local government (*p* = 0.001) among the occupations; however, there was no significant difference in unmet support needs.

**Table 2:** The received support, support needs, and unmet support needs of home health care workers during the COVID-19 pandemic

	All (*n* = 311)	%	Doctor (*n* = 77)	%	Nurse (*n* = 102)	%	Medical social worker (*n* = 21)	%	Medical office staff (*n* = 56)	%	Other occupations (*n* = 56)	%	*p*
We have been provided with expert on-site infection control guidance													
Yes	64	20.6	14	18.2	22	21.6	6	28.6	11	19.6	11	19.6	0.875
Need for experts to provide on-site infection control guidance													0.099
Not needed at all/Not needed/Little need	32	10.3	10	13.0	8	7.8	1	4.8	7	12.5	6	10.7	
Some need	102	32.8	24	31.2	27	26.5	11	52.4	24	42.9	16	28.6	
Very much needed/Needed	166	53.4	41	53.2	65	63.7	8	38.1	21	37.5	30	53.6	
Unmet support need for experts to provide on-site infection control guidance													
Yes	122	39.2	31	40.3	48	47.1	4	19.0	15	26.8	23	41.1	0.059
We have a system to consult with infection control experts online or by phone when necessary													
Yes	73	23.5	22	28.6	28	27.5	2	9.5	8	14.3	13	23.2	0.183
Need for system to consult with infection control experts online or by phone when necessary													0.059
Not needed at all/Not needed/Little need	18	5.8	1	1.3	3	2.9	3	14.3	7	12.5	4	7.1	
Some need	109	35.0	30	39.0	33	32.4	6	28.6	21	37.5	19	33.9	
Needed/Very much needed	173	55.6	44	57.1	64	62.7	11	52.4	24	42.9	29	51.8	
Unmet support need for systems to consult with infection control experts online or by phone													
Yes	119	38.3	27	35.1	44	43.1	9	42.9	18	32.1	20	35.7	0.739
We are able to attend expert lectures on COVID-19													
Yes	89	28.6	40	51.9	29	28.4	7	33.3	4	7.1	9	16.1	< 0.001
The need for the expert lectures on COVID-19													0.002
Not needed at all/Not needed/Little need	19	6.1	2	2.6	5	4.9	3	14.3	6	10.7	3	5.4	
Some need	110	35.4	19	24.7	34	33.3	5	23.8	28	50.0	24	42.9	
Needed/Very much needed	171	55.0	54	70.1	61	59.8	12	57.1	18	32.1	25	44.6	
Unmet support need for expert lectures on COVID-19													
Yes	100	32.2	19	24.7	37	36.3	6	28.6	16	28.6	21	37.5	0.385
We have a system of distribution of personal protective equipment by national or local government													
Yes	136	43.7	38	49.4	55	53.9	6	28.6	24	42.9	12	21.4	0.002
Need for system of distribution of personal protective equipment by national or local government													0.001
Not needed at all/Not needed/Little need	26	8.4	4	5.2	2	2.0	4	19.0	6	10.7	10	17.9	
Some need	62	19.9	11	14.3	21	20.6	5	23.8	17	30.4	8	14.3	
Needed/Very much needed	212	68.2	60	77.9	77	75.5	11	52.4	29	51.8	34	60.7	
Unmet support need for system of distribution of PPE by national or local government													
Yes	102	32.8	26	33.8	32	31.4	8	38.1	12	21.4	24	42.9	0.155
We have systems to support our psychological stress and emotional exhaustion													
Yes	81	26.0	22	28.6	23	22.5	8	38.1	9	16.1	19	33.9	0.107
Need for systems to support our psychological stress and emotional exhaustion													0.358
Not needed at all/Not needed/Little need	29	9.3	4	5.2	11	10.8	2	9.5	5	8.9	7	12.5	
Some need	93	29.9	29	37.7	28	27.5	3	14.3	20	35.7	13	23.2	
Needed/Very much needed	178	57.2	42	54.5	61	59.8	15	71.4	27	48.2	32	57.1	
Unmet support need for systems to support our psychological stress and emotional exhaustion													
Yes	118	37.9	24	31.2	44	43.1	8	38.1	22	39.3	19	33.9	0.566

The mean score of the FCV-19S-J was 16.5 ± 5.0 (7.0 – 31.0), HADS-anxiety was 5.3 ± 3.3 (0.0 – 18.0), and HADS-depression was 6.5 ± 3.6 (0.0 – 16.0). The rates of definitive anxiety and depression were 7.4% and 15.7, respectively. The mean FCV-19S-J scores of nurses, medical office staff, and other occupations were significantly higher than those of doctors. In addition, the mean scores of HADS-depression for nurses, medical social workers, medical office staff, and other occupations were significantly higher than those of doctors (Table 3).

**Table 3:** Descriptive statistics and one-way ANOVA of FCV-19S-J and HADS

	All (*n* = 311)	Doctors (*n* = 77)	Nurses (*n* = 102)	p*	Medical social workers (*n* = 21)	p*	Medical office staff (*n* = 56)	p*	Other occupations (*n* = 56)	p*
FCV-19S-J										
mean ± standard deviation	16.5 ± 5.0	14.5 ± 4.6	17.2 ± 5.1	0.003	16.1 ± 5.3	0.699	18.2 ± 4.5	< 0.001	16.4 ± 5.0	0.200
median (min, max)	17.0 (7.0, 31.0)	14.0 (7.0, 24.0)	17.0 (7.0, 31.0)		14.5 (7.0, 27.0)		18.0 (8.0, 29.0)		17.0 (7.0, 28.0)	
HADS										
Anxiety										
mean ± standard deviation	5.3 ± 3.3	4.4 ± 3.1	5.6 ± 3.5	0.103	6.3 ± 3.5	0.145	5.7 ± 3.4	0.171	5.1 ± 2.8	0.782
median (min, max)	5.0 (0.0, 18.0)	4.0 (0.0, 14.0)	5.0 (0.0, 18.0)		6.0 (1.0, 16.0)		5.0 (1.0, 16.0)		4.0 (0.0, 12.0)	
≥ 11	7.4%	4.0%	8.0%		10.0%		11.8%		5.8%	
≥ 8	21.1%	12.0%	24.0%		20.0%		25.5%		21.2%	
Depression										
mean ± standard deviation	6.5 ± 3.6	4.7 ± 3.1	7.4 ± 3.8	< 0.001	7.7 ± 3.6	0.007	7.2 ± 3.4	< 0.001	6.5 ± 3.3	0.031
median (min, max)	6.0 (0.0, 16.0)	5.0 (0.0, 12.0)	8.0 (0.0, 16.0)		7.0 (2.0, 15.0)		7.0 (1.0, 15.0)		6.0 (0.0, 14.0)	
≥ 11	15.7%	4.0%	26.0%		30.0%		13.7%		13.5%	
≥ 8	38.8%	17.3%	52.0%		40.0%		41.2%		42.3%	

^*^p: compared with doctors, tested with Tukey’s HSD

Multivariate regression analysis of FCV-19S-J, HADS-anxiety, and HADS-depression revealed that the J-AITCS-II teamwork subscale was significantly negatively associated with FCV-19S-J, HADS-anxiety, and HADS-depression (β = -0.171, *p* = 0.004; β = -0.151, *p* = 0.012; β = -0.225, *p* < 0.001, respectively). Medical office staff showed a significant positive association with FCV-19S-J and HADS-depression (β = 0.219, *p* = 0.005; β = 0.201, *p* = 0.009, respectively), and medical social workers showed significant positive associations with HADS-anxiety and HADS-depression compared with doctors (β = -0.166, *p* = 0.011; β = -0.214, *p* < 0.001, respectively). The unmet support need for expert lectures on COVID-19 was significantly positively associated with FCV-19S-J (β = 0.131, *p* = 0.048), and the unmet support need for systems to support psychological stress and emotional exhaustion was significantly positively associated with HADS-anxiety (β = 0.141, *p* = 0.022) (Table 4).

**Table 4:** Multivariate regression analysis of FCV-19S-J and HADS

	FCV-19S-J		*p*	HADS- anxiety		*p*	HADS- depression		*p*
	β	95% CI		β	95% CI		β	95% CI	
Female	0.084	-.0.68—2.50	0.262	-0.016	-1.20—0.96	0.832	0.062	-0.64—1.62	0.395
Age over 60	0.037	-0.79—1.58	0.514	-0.004	-0.83—0.78	0.950	0.093	-0.13—1.55	0.099
Type of occupation									
ref: Doctor	-	-	-	-	-	-	-	-	-
Nurse	0.168	-0.05—3.58	0.056	0.157	-0.13—2.33	0.081	0.282	0.86—3.45	0.001
Medical social worker	0.094	-0.62—4.31	0.142	0.166	0.50—3.85	0.011	0.214	1.30—4.82	< 0.001
Medical office staff	0.219	0.86—4.92	0.005	0.124	-0.30 -2.47	0.123	0.201	0.48—3.38	0.009
Others	0.136	0.02—3.56	0.047	0.071	-0.58—1.82	0.310	0.150	0.18—2.69	0.026
J-AITCS-II teamwork subscale	-0.171	-0.25—-0.05	0.004	-0.151	-0.16—-0.02	0.012	-0.225	-0.22—-0.07	< 0.001
Increased workload	0.108	-0.05—2.85	0.059	0.067	-0.41—1.56	0.254	-0.012	-1.14—0.93	0.836
Unmet support needs									
experts to provide on-site infection control guidance	0.023	-1.06—1.53	0.723	0.014	-0.79—0.98	0.833	-0.049	-1.29—0.56	0.438
system to consult with infection control experts online or by phone	-0.024	-1.53—1.04	0.707	-0.067	-1.33—0.42	0.305	0.043	-0.60—1.23	0.498
expert lectures on COVID-19	0.131	0.01 0 2.76	0.048	0.138	0.04—1.91	0.041	0.064	-0.49—1.47	0.324
system of distribution of PPE by national or local government	-0.035	-1.61—0.89	0.566	-0.100	-1.55—0.15	0.106	-0.062	-1.36—0.42	0.300
system to support psychological stress and emotional exhaustion	0.086	-0.32—2.08	0.149	0.141	0.14—1.77	0.022	0.039	-0.56—1.14	0.503

## Discussion

To the best of our knowledge, this is the first large-scale nationwide survey to investigate the status and associated factors of fear of COVID-19 infection and mental health among home-HCWs. A notable finding of our study was that the type of occupation, teamwork, and unmet support needs were associated with the fear of COVID-19 infection and the mental health of home-HCWs.

Our study found that the FCV-19S-J scores of home-HCWs were similar to those in the general Japanese population in August 2020. Also, our study found that the prevalence of anxiety and depression in home-HCWs was relatively low compared to front-line health care workers in hospitals [9, 20, 25, 27]. These results suggest that the mental health condition of HCWs may differ based on the setting, irrespective of the pandemic.

Our study revealed that the FCV-19S-J scores of nurses, medical office staff, and other occupations were significantly higher than those of doctors. In addition, the HADS-depression scores of nurses, medical social workers, medical office staff, and other occupations were significantly higher than those of doctors. These findings suggest that non-physicians are more likely to be fearful and depressed during the COVID-19 pandemic and that tailored mental health support based on occupation, especially for non-physicians, is needed in the home care setting. Furthermore, Babicki et al. revealed that the experience of being made to take a secondment to work with COVID-19 patients, which differed among country and epidemic situations, had a significant impact on the mental health status of health care professionals [8]. Further studies of the influence of being made to take a secondment to work with COVID-19 patients on the mental health status of home-HCWs is needed in the future.

Multivariate regression analysis revealed that medical office staff had a significant positive association with FCV-19S-J score, and medical social workers had a significant positive association with HADS-anxiety. As our study found that medical office work and medical social work are possible risk factors for fear of COVID-19 infection and deterioration of mental health in the home care setting, our findings support a previous study that found that nurses or front-line workers had a high prevalence of fear of COVID-19 infection and anxiety [10]. A possible reason for our findings is that medical office staff and medical social workers do not have sufficient knowledge about COVID-19 infection. Another possible reason is that as both medical office staff, who interact with patients and/or families, and medical social workers, who conduct the first intake with patients and/or families, are often the first contact person in the home care setting, the unknown risk of infection may worsen their fear of COVID-19 infection and their mental health condition. Our findings highlight the need for appropriate infection protection systems and mental health support for non-physicians and non-nursing staff as well as physicians and nurses during the COVID-19 pandemic.

Perceived good teamwork within each facility had a significant inverse association with the fear of COVID-19 scale and HADS-anxiety and depression. This result is partially consistent with a previous systematic review that reported that dissatisfaction with teamwork was significantly associated with depression but not with both anxiety and stress among nurses [28]. Thus, our study presents novel evidence about the mental health of home-HCWs during the COVID-19 pandemic in that good teamwork can mitigate the negative influence on home-HCWs’ mental health regardless of the type of occupation. In a study conducted before the COVID-19 pandemic, interprofessional factors, such as teamwork, collaboration, and cooperation, were positively associated with job satisfaction, and dissatisfaction with teamwork was significantly associated with depression among expatriate nurses [28]. However, a theoretical foundation for this association between interprofessional work and mental health of HCWs during the COVID-19 pandemic is still being developed [29]. Our findings suggest that it is important for an organization to regularly promote teamwork to improve the quality of care for patients and to maintain and improve the mental health of staff. Our study may contribute to theory-based interprofessional work in the home care setting in the COVID-19 era.

Our study found that it is important to provide home-HCWs with learning opportunities about emerging infectious diseases and psychological support from experts to maintain and improve the mental health of the staff during the COVID-19 pandemic. In addition, as described in a previous systematic review, these efforts can be carried out as daily activities based on the needs of the staff, which could enhance teamwork [30].

We found significant differences in support needs among the types of occupation during the COVID-19 pandemic; however, there were no significant differences in unmet support needs. This finding suggests that support needs during the COVID-19 pandemic may vary by type of occupation, and unmet support needs may vary by facility initiatives. It is important for facility managers to understand the support needs of their staff and to provide systematic support according to individual needs. In addition, this result suggests that the authorities, which tend to focus on providing support to physicians who directly care for COVID-19 patients, need to provide more support to other health care professionals working in the home care setting.

The strengths of our study included a relatively large sample size and multidisciplinary participants nationwide. Our study had some limitations. First, we used variables that have not been validated, although we measured the outcomes with well-validated scales. Second, our study was a cross-sectional survey and could not determine causality among the outcomes and variables. Third, as we did not ask how many staff were candidates for this survey at each facility, we could not calculate the response rate. Therefore, the generalizability of the results should be interpreted with caution. Fourth, we did not assess the presence of anxiety and/or depression of the participants before the COVID-19 pandemic. Fifth, the results may have been influenced by the situation of the pandemic; thus, we were unable to draw definitive conclusions about associated factors of anxiety and/or depression during the COVID-19 pandemic. We believe that these limitations are unlikely to have had a significant impact on the results of our study, and that this study drew on the best available evidence during the COVID-19 pandemic.

## Conclusions

The fear of COVID-19 infection and depression of nurses, medical office staff, and other occupations were significantly higher than those of doctors. These findings suggest that non-physicians are more likely to be fearful and depressed during the COVID-19 pandemic. Tailored mental health support based on occupation is needed in the home care setting.

## Data Availability

The datasets generated and analyzed during the current study are not publicly available due to them containing information that could compromise research participant privacy/consent, but are available from the corresponding author on reasonable request.

## Abbreviations

home-HCWs: Home health care workers
PPE: Personal protective equipment
J-AITCS-II: Japanese version of Assessment of Interprofessional Team Collaboration Scale-II
AITCS-II: Shortened version of Assessment of Interprofessional Team Collaboration Scale
FCV-19S-J: Japanese version of the Fear of COVID-19 scale
FCV-19S: Fear of COVID-19 scale
HADS: Hospital Anxiety and Depression scale
